# Emodin Inhibits EBV Reactivation and Represses NPC Tumorigenesis

**DOI:** 10.3390/cancers11111795

**Published:** 2019-11-15

**Authors:** Chung-Chun Wu, Mei-Shu Chen, Yu-Jhen Cheng, Ying-Chieh Ko, Su-Fang Lin, Ing-Ming Chiu, Jen-Yang Chen

**Affiliations:** 1 National Institute of Cancer Research, National Health Research Institutes, No. 35, Keyan Road, Zhunan Town 350, Taiwan; 2 Institute of Cellular and System Medicine, National Health Research Institutes, Zhunan Town 350, Taiwan; 3 Department of Microbiology, College of Medicine, National Taiwan University, Taipei 100, Taiwan

**Keywords:** emodin, Epstein-Barr virus, reactivation, nasopharyngeal carcinoma, relapse

## Abstract

Nasopharyngeal carcinoma (NPC) is a unique malignancy derived from the epithelium of the nasopharynx. Despite great advances in the development of radiotherapy and chemotherapy, relapse and metastasis in NPC patients remain major causes of mortality. Evidence accumulated over recent years indicates that Epstein-Barr virus (EBV) lytic replication plays an important role in the pathogenesis of NPC and inhibition of EBV reactivation is now being considered as a goal for the therapy of EBV-associated cancers. With this in mind, a panel of dietary compounds was screened and emodin was found to have potential anti-EBV activity. Through Western blotting, immunofluorescence, and flow cytometric analysis, we show that emodin inhibits the expression of EBV lytic proteins and blocks virion production in EBV- positive epithelial cell lines. In investigating the underlying mechanism, reporter assays indicated that emodin represses Zta promoter (Zp) and Rta promoter (Rp) activities, triggered by various inducers. Mapping of the Zp construct reveals that the SP1 binding region is important for emodin-triggered repression and emodin is shown to be able to inhibit SP1 expression, suggesting that it likely inhibits EBV reactivation by suppression of SP1 expression. Moreover, we also show that emodin inhibits the tumorigenic properties induced by repeated EBV reactivation, including micronucleus formation, cell proliferation, migration, and matrigel invasiveness. Emodin administration also represses the tumor growth in mice which is induced by EBV activation. Taken together, our results provide a potential chemopreventive agent in restricting EBV reactivation and NPC recurrence.

## 1. Introduction

Nasopharyngeal carcinoma (NPC) is a squamous cell carcinoma rising at the post nasal cavity, which is prevalent in southern China, southeastern Asia, and Taiwan. Worldwide, approximately 80,000 cases of NPC are reported each year, 0.7% of all cancers [1]. In general, the 5-year survival rate of NPC is 60%. When treatment begins at an early stage, the 5-year survival rate can reach 80–95%; however, it is poor at a later stage of NPC [2]. Radiotherapy is the primary and effective treatment for NPC. The combination of radiotherapy with neoadjuvant chemotherapy is another pivotal treatment for NPC patients and increases the survival rate significantly [3,4,5]. Although the therapeutic efficiency of NPC management has largely been improved, how to avoid NPC metastasis is still an urgent unmet need. 

Genetic, environmental, and viral factors have been incriminated in the etiology of NPC and Epstein-Barr virus (EBV) is strongly associated with the occurrence of NPC. EBV is a member of the gamma herpesviruses, with a 172 kb double-stranded DNA genome. The life cycle of EBV includes latency and lytic replication. Eleven gene products, EBNAs 1~6, LMPs 1, 2A, and 2B, and small RNAs EBER 1 and 2, are expressed during latent infection [6]. Upon induction by chemicals or stress, EBV is activated and enters the lytic cycle, known as reactivation. Three subsets of lytic genes, immediate-early, early, and late genes, are expressed sequentially and, subsequently, viral particles are packaged and released [7,8]. Through years of study, EBV latent infection has been considered to play an important role in NPC carcinogenesis. In recent years, accumulating evidence has suggested that EBV lytic infection also contributes to the tumorigenesis of NPC. Sero-epidemiological surveys indicate that patients with elevated antibody titers against EBV lytic proteins, such as viral capsid antigen (VCA), DNase, DNA polymerase, and early antigen (EA), have a high risk of NPC [9,10,11,12,13,14,15]. Several EBV lytic products have been detected in the NPC biopsies [16,17,18]. In addition, EBV lytic proteins, including BARF1, BHRF1, BRLF1, BALF3, BGLF4, and DNase, have been shown to have various tumorigenic functions [16,19,20,21,22]. In our previous study, we found that EBV reactivated by chemicals promotes cellular genome instability and tumor growth [23,24]. Collectively, these reports suggest that inhibition of EBV reactivation might be useful for NPC prevention and therapy.

Accordingly, for safety and convenience, we have screened several dietary compounds for anti-EBV activity. We reported that the flavonoids have activity against EBV reactivation and through the inhibition of EBV, NPC tumorigenesis has been repressed [25,26,27]. Here, we report that the phytochemical emodin has the potential ability to inhibit EBV reactivation.

Emodin, a common traditional Chinese medicine, is an anthraquinone derivative present in the roots or rhizomes of rhubarb. Emodin has various medical applications, including anti-cancer, anti-oxidation, anti-inflammation or allergy, anti-diabetes, and anti-viral or bacterial activities [28]. For its anti-viral effect, emodin has the ability to inhibit the SARS coronavirus [29], and block the replication of HBV [30], Coxsackievirus [31], zika virus [32], and influenza A virus [33]. Moreover, emodin has been suggested to inhibit the replication of herpesviruses [34,35,36]. However, the detailed mechanism is not well elucidated. In this study, based on our screening, we show that emodin inhibits EBV reactivation in epithelial cells, through the mechanism of repressing SP1 expression to inhibit the Zta and Rta promoter activities. Moreover, through its anti-EBV activity, NPC tumorigenesis could be relieved, suggesting emodin is a potential agent for therapy of EBV-associated cancer.

## 2. Results

### 2.1. The Cytotoxicity of Emodin to the NPC Cell Lines

**Figure 1:**
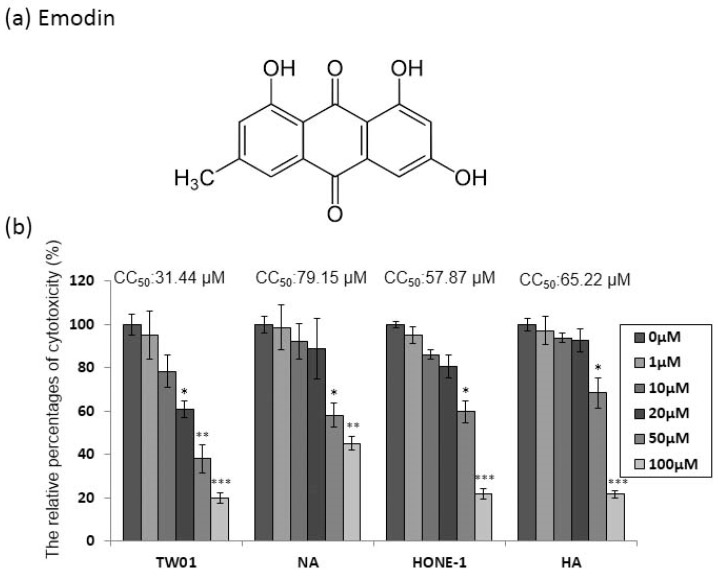
Epstein-Barr virus (EBV) positive nasopharyngeal carcinoma (NPC) cells are more resistant to emodin. (**a**) The chemical structure of emodin. (**b**) NPC cell lines (TW01, HONE-1) and their EBV infected counterparts (NA, HA) were treated with indicated concentrations of emodin for 48 h, followed by cell viability assay and CC50 calculation (top of each panel). The values are means ± SD from at least three independent experiments. (* *p* < 0.05, ** *p* < 0.01, *** *p* < 0.001 compared to the group of 0 μM).

Emodin (1,3,8-trihydroxy-6-methyl-anthraquinone, Figure 1a) has been shown to be cytotoxic to human cell lines. Before investigating the anti-EBV effect of emodin, two EBV-positive NPC cell lines, NA and HA, and their parental cells TW01 and HONE-1, were assessed for susceptibility to the cytotoxicity of emodin. The cells were seeded into 96-well plates 24 h prior to treatment; then, emodin was added for 48 h to determine its cytotoxic effect on the NPC cell lines NA and HA. Emodin has significant toxicity to TW01 cells, while the derivative NA cells showed little resistance to the compound (Figure 1b). A similar effect was seen on HONE-1 and HA cells (Figure 1b). Furthermore, to evaluate these results more precisely, half maximum of cytotoxicity concentrations 50 (CC50) were calculated and are shown in the top panels of Figure 1b. The CC50 values of TW01 and the derivative NA cell lines were 31 and 79 μM, while the values for HONE-1 and HA were 58 and 65 μM, respectively (Figure 1b), implying that the cells harboring EBV seemed to be more resistant to emodin (TW01 vs. NA: *p* < 0.01; HONE1 vs. HA: *p* = 0.06). Based on these results, we chose 1 to 50 μM of emodin as our working concentrations for further studies.

### 2.2. Emodin Inhibits EBV Lytic Protein Expression in NPC Cells

**Figure 2:**
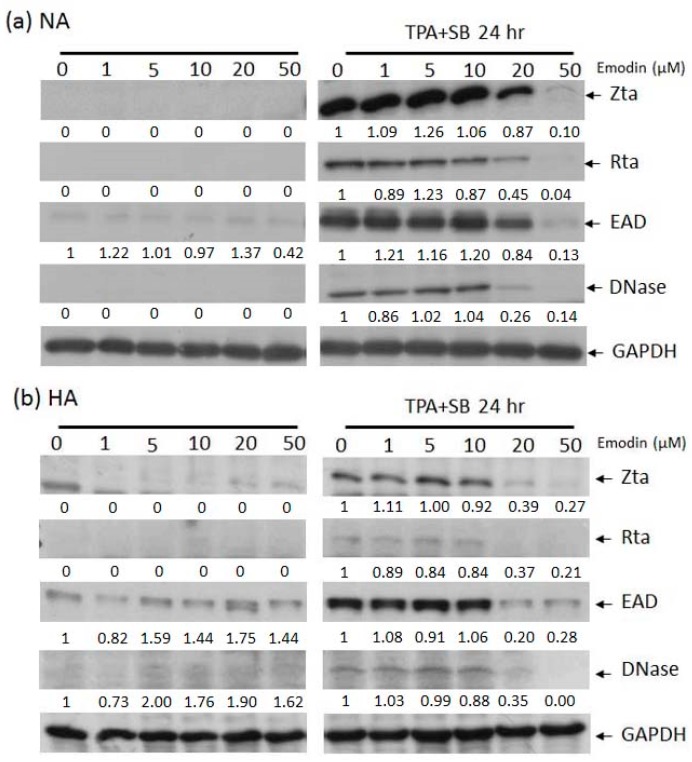
The expression of EBV lytic proteins in epithelial cell lines is inhibited by emodin. Western blot analysis of EBV positive NA (**a**) and HA (**b**) cells treated with emodin alone (left panels) or emodin and TPA+SB (right panels). Zta, Rta, EAD, DNase are EBV lytic proteins, GAPDH serves as a loading control. Forty ng/mL TPA, 3 mM SB, and 1 to 50 μM emodin were used in this experiment.

In our hands, EBV lytic replication can be efficiently induced by treating NA or HA cells with 40 ng/mL 12-*O*-tetradecanoyl-phorbol-1,3-acetate (TPA) plus 3 mM sodium butyrate (SB) (TPA+SB) for 24 h. To determine whether emodin has effects on EBV reactivation, prior to TPA+SB induction, cells were exposed to various concentrations of emodin for 1 h, followed by Western blot analysis of EBV lytic proteins Zta, Rta, EAD, and DNase. As shown in Figure 2, no lytic protein expression was induced by emodin alone in NA nor HA cells (left panels). However, when cells were co-treated with emodin, the expression of lytic proteins was decreased in a dose-dependent manner, suggesting that emodin has the ability to inhibit EBV reactivation in NPC cells (right panels).

**Figure 3:**
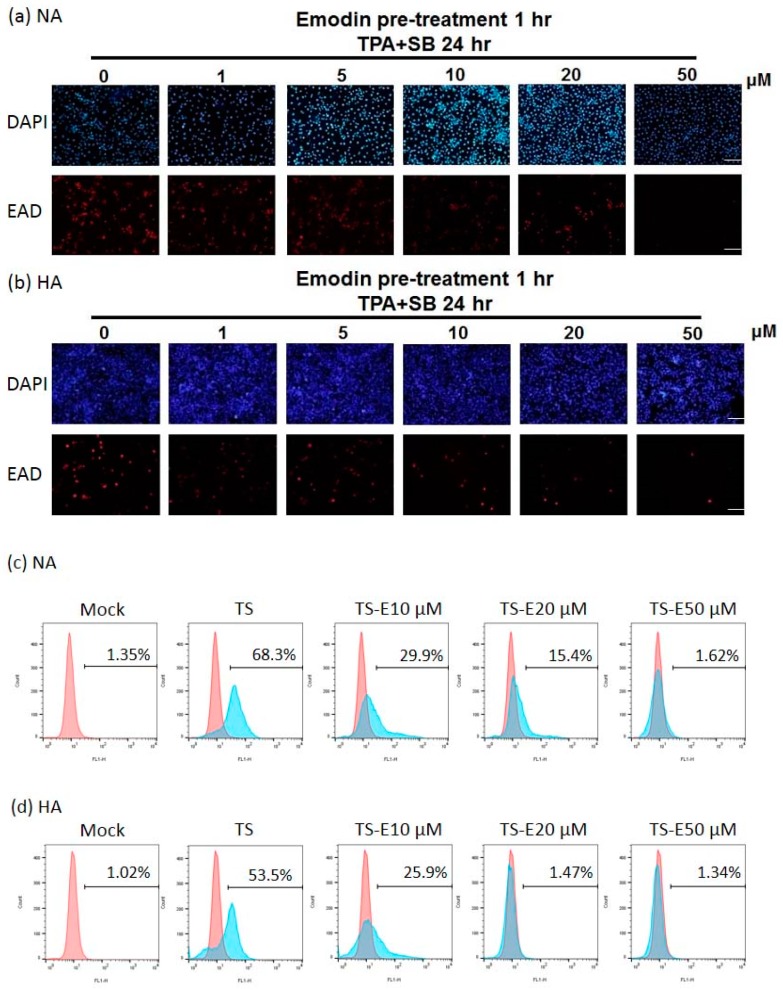
The amount of EAD-positive cells is decreased by emodin treatment of EBV-positive epithelial cell lines. (**a**,**b**) immunofluorescence assay of NA (**a**) and HA (**b**) cells exposed to indicated concentrations of emodin one hour before TPA+SB induction (TPA+SB 24 h). EBV diffusive early antigen (EAD) serves as a surrogate marker for virus reactivation (red). Scale bar: 100 μm. (**c**,**d**) Flow cytometry analysis was used to quantitate EAD positive cells in each treatment as denoted in (**a**,**b**). TS, TPA + SB; E, emodin.

To confirm the inhibitory effect of emodin on EBV, the population of cells expressing EAD as a marker of EBV reactivation was detected by immunofluorescence staining to monitor the inhibition of reactivation by emodin. Emodin could not induce EAD expression in the NA and HA cell lines, however, the population of EAD-expressing NA (Figure 3a) and HA (Figure 3b) cells decreased gradually following treatment with increasing concentrations of emodin. Emodin repressed the numbers of EAD-expressing NA and HA cells moderately at a dose of 10 μM and significantly at 20 μM with complete blockage at 50 μM.

We quantified the changes of the proportion of EAD-positive cells by flow cytometry after EBV induction, followed by emodin treatment. With 24 h induction by TPA + SB, the percentage of EAD-positive NA cells was 68%, while this reduced to 30% and 15% after treatment with 10 μM and 20 μM emodin, respectively (Figure 3c). At the meantime, 54% of HA cells expressed EAD after TPA + SB induction (Figure 3d), which was lower than NA cells (68%). The proportions of EAD-expressing HA cells were 26% and 1% following treatment with 10 and 20 μM of emodin, respectively (Figure 3d). These results give another evidence to support our hypothesis that emodin can inhibit the EBV lytic cycle.

### 2.3. The Inhibition of Virion Production by Emodin

**Figure 4:**
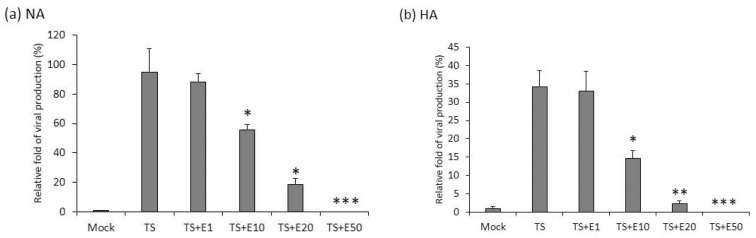
Virus production is inhibited by emodin treatment. EBV virions released in the culture media of NA (**a**) and HA (**b**) cells treated with TPA + SB (TS) or TS plus emodin (TS + E1, TS + E10, TS + E20, TS + E50) were collected, treated with DNase I to remove free DNA present in the media. Viral genome was purified by proteinase K digestion and subjected to qPCR analysis of EBV DNA polymerase gene fragment (BALF5). Untreated cells served as spontaneous lytic replication control (Mock). Data are means ± SD of two independent experiments. (* *p* < 0.05, ** *p* < 0.01, *** *p* < 0.001 compared to the TS group).

After we demonstrated that emodin blocks the expression of EBV lytic proteins, a further question was raised: can emodin inhibit the EBV lytic cycle completely? To solve this issue, NPC cells were pre-treated with emodin for 1 h, followed by TPA + SB treatment. After incubation for 48 h, the amounts of released EB virions were measured in the supernatants. Emodin has the ability to inhibit virion production by EBV in a dose-dependent manner in both NA (Figure 4a) and HA (Figure 4b) cells.

Taken together, the results above indicate that emodin can repress EBV lytic protein expression and attenuate virion production, clearly suggesting its ability to inhibit EBV reactivation.

### 2.4. The Repression of Zta Promoter (Zp) and Rta Promoter (Rp) Transcriptional Activities by Emodin

**Figure 5:**
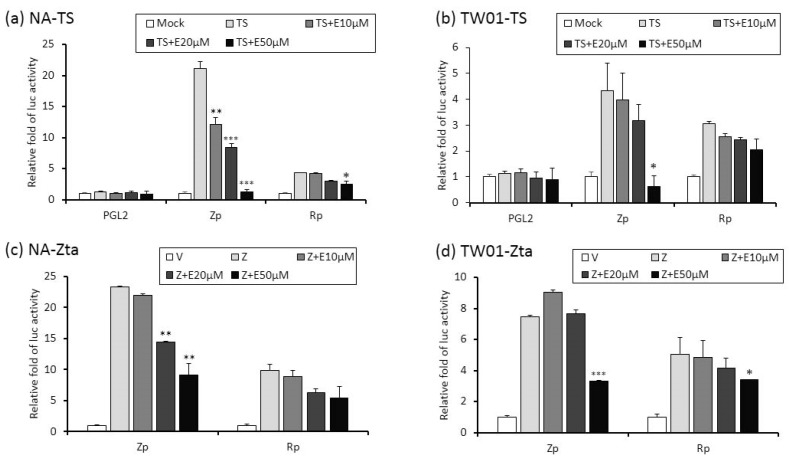

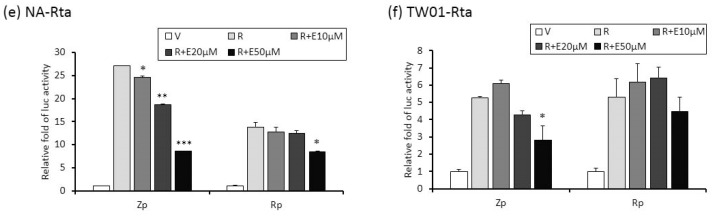
The activities of Zp and Rp are repressed by emodin treatment of NA cells. (**a**,**b**) NA and its parental EBV negative cell line, TW01, were transfected with luciferase reporters containing Zp or Rp, followed by emodin (E) and TPA + SB (TS) treatments. After TS induction for 24 h, cell lysates were collected for measurement of luciferase activity. Data are means ± SD from at least two independent experiments. (**c**,**d**) Zta-expressing plasmid (Z) was co-transfected with Zp or Rp luciferase reporters into NA (**c**) or TW01 (**d**) cells, with same emodin and TPA+SB treatments depicted in (**a**,**b**). (**e**,**f**) same to (**c**,**d**), except Rta-expressing plasmid (R) was used. (* *p* < 0.05, ** *p* < 0.01, *** *p* < 0.001 compared to the groups of TS, Z or R, respectively).

Zta and Rta are two important immediate-early (IE) proteins involved in the initiation of EBV lytic reactivation. To access whether emodin exerts its anti-EBV activity through interfering with IE gene promoters, a luciferase reporting assay was performed to detect promoter activities (Zp and Rp, respectively) in the presence or absence of emodin. Both EBV-positive (NA) and -negative (TW01) NPC cells were used in this study. As shown in Figure 5a,b, while TPA+SB significantly increased Zp and Rp activities in both NA and TW01 cells, addition of emodin decreased both promoter activities in a dose-dependent manner. Of note, promoter activities detected in NA cells are higher than in TW01 cells because the EBV harboring in NA cells creates an autocrine regulation to amplify the Zp and Rp activities under simulation. Next, in addition to TPA + SB, we asked whether emodin also inhibits Zta or Rta mediated EBV reactivation. To this end, Zta- or Rta-expressing plasmids were co-transfected with Zp or Rp reporter plasmids, respectively, followed by emodin treatment for 24 h. As expected, ectopic Zta activated both Zp and Rp, whereas co-treatment of emodin significantly reduced both promoter activities in a dose-dependent manner (Figure 5c,d). Similarly, over-expression of Rta resulted in Zp and Rp activation; addition of emodin reversed this phenomenon (Figure 5e,f). Thus, these results suggest that emodin is able to inhibit both chemical and Zta/Rta-induced EBV lytic reactivation via repressing IE gene promoter activation.

### 2.5. Identification of Emodin Responsive Element in Zp

**Figure 6:**
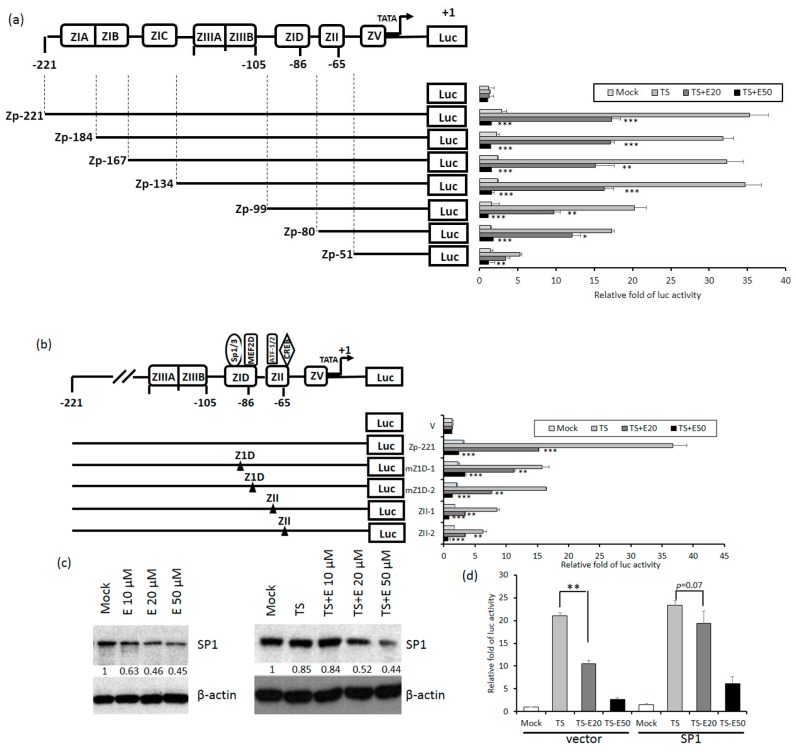
Z1D and ZII domains of Zp are important for emodin inhibition. (**a**) Luciferase activities of TW01 cells harboring wild type Zp (spanning −221 to +1 region, top) or serial 5’-deletion mutants (Zp-184, −167, −134, −99, −80, −51). Three hours after transfection, the cells were pre-treated with emodin for 1 h followed by TPA+SB treatment for an additional 24 h. The relative folds of luciferase activities are means ± SD from three independent experiments. (**b**) Luciferase activities of emodin/TPA+SB-treated TW01 cells harboring wild type Zp and mutants of cellular factor binding sites located in the Z1D and ZII domains (mZ1D-1, mZ1D-2, ZII-1, ZII-2). The relevant mutated sites are represented by a black triangle (▲). (**c**) The SP1 and β-actin of NA cells were detected after EBV induction with emodin treatment for 24 h. (**d**) NA cells were co-transfected with Zp reporter and vector or SP1-expressing plasmid for 3 h, respectively. After transfection, the cells were pretreated with emodin and activated EBV with TPA+SB. After 24 h of incubation, the luciferase activities were detected. Values are means ± SD from three independent experiments. (* *p* < 0.05, ** *p* < 0.01, *** *p* < 0.001 compared to the group of TS).

Because Zta is the first protein expressed in the EBV lytic cycle, we wished to know which elements in Zp are essential for emodin inhibition. To this end, luciferase-reporting plasmids driven by a series of 5’-deletion mutants of Zp were constructed. These mutants were respectively transfected into TW01 cells, followed by aforementioned compound treatment and luciferase-reporting assay. As depicted in Figure 6a, compared to wild type, promoter Zp-99 retained only half of the activity; promoters shorter than this region (Zp-80, Zp-51) gradually lose their responsiveness to TPA+SB. Notably, the inhibition of Zp activity by 20 and 50 μM emodin also showed a similar pattern, indicating that elements required for emodin inhibition reside in region −99 to −51. There are two major domains located within this region, Z1D and ZII. Z1D is known to be involved in SP1/SP3 and MEF2D regulation; ZII contains several transcriptional factor binding sites important for Zp activation, including ATF-1, ATF-2, and CREB. To determine which regulatory factor is important for emodin inhibition, four Zp constructs with mutations in Z1D and ZII domains, designated as mZ1D-1, mZ1D-2, ZII-1, and ZII-2, respectively, were used for compound treatment and luciferase-reporting assay mentioned above. As shown in Figure 6b, emodin inhibition phenomenon was compromised significantly in mZ1D-1, mZII-1, and mZII-2, whereas the activity of mZ1D-2 maintained a similar level to the wild-type control. Among them, the inhibition fold in mZ1D-1 displayed a significant alteration and attracted our attention. We sought to determine whether emodin affects the expression of SP1. As shown in Figure 6c, SP1 expression was repressed by emodin in a dose-dependent manner with or without TPA+SB co-treatment, implying emodin inhibits Zp activity by repressing SP1 expression. Finally, we sought to identify whether emodin-elicited Zp inhibition is mediated by SP1 repression. We transfected SP1-expressing plasmid to resupply the SP1 protein level under the emodin treatment and determined whether the Zp activity can be compensated. The results revealed that TPA+SB-activated Zp activity was repressed by emodin significantly (Figure 6d, left panel), however, this repression was compensated gradually when resupplied with SP1 (Figure 6d, right panel). To sum up, these results suggested that emodin inhibits EBV reactivation through SP1 repression.

### 2.6. Emodin Attenuates the Reactivation-Induced Tumorigenic Properties of NPC Cells

**Figure 7:**
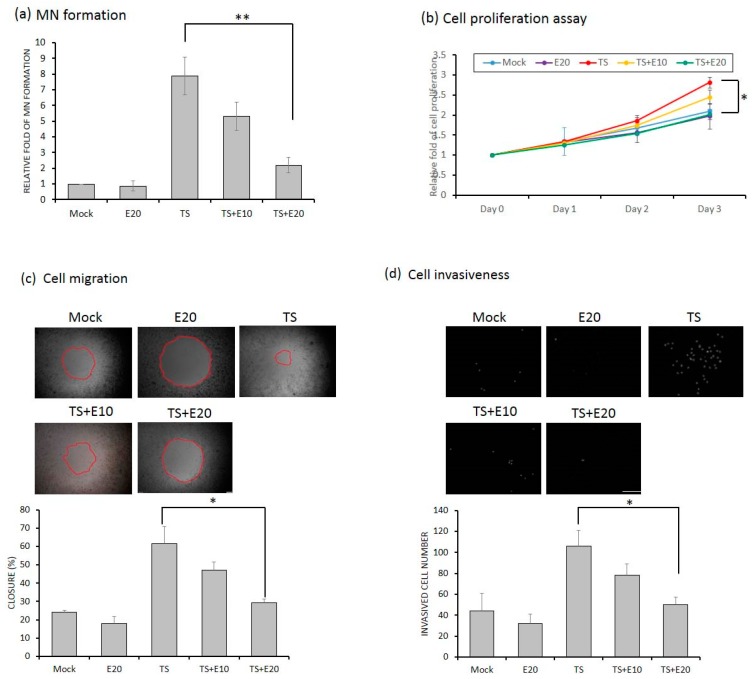
Emodin represses reactivation-induced tumorigenic properties. The EBV-positive cell line NA was treated with TPA+SB repeatedly to investigate various tumorigenic properties. (**a**) For detection of MN formation, the cells were harvested after 10 repeated treatments with TPA+SB, with or without emodin, and stained with DAPI for MN examination using fluorescence microscopy. (**b**) For detection of cell proliferation, the cells were subjected to WST-1 assay after 10 repeated treatments with TPA+SB, with or without emodin, to detect the cell proliferation over the following 3 days. For cell migration (**c**) and cell invasion (**d**) assays, repeated TPA+SB induced NA cells were subjected to Oris system (c) or HTS FluoroBlock transwell (d) in the presence or absence of emodin, followed by cell counts. Scale bar: 100 μm. Data are the mean ± SD from three independent experiments. * *p* < 0.05; ** *p* < 0.01.

Previously, we showed that recurrent EBV reactivation leads to more profound genomic instability and NPC tumorigenesis [23,24]. Importantly, these effects would be prevented if EBV reactivation could be blocked [26]. To test whether emodin can inhibit the EBV reactivation-induced tumorigenic properties, the cell culture model with repeated EBV reactivation combined with emodin treatment was used to examine the impact of emodin on the reactivation-induced malignant characteristics of NA cells. First, 10 repeated TS-induced NA cells with or without emodin treatment were subjected to micronucleus (MN) formation assay. As expected, we observed emodin affectively and does-dependently decreased MN formation, a prominent feature associated with EBV reactivation (Figure 7a). Next, since EBV reactivation has been implicated in several tumorigenic properties of NPC cells, including cell proliferation, cell migration, and cell invasion [24,26], we sought to determine whether emodin suppresses these malignant properties induced by recurrent EBV reactivation. As shown in Figure 7b, an increase of cell proliferation was observed in the TS-treated group. This increase was gradually reduced with emodin treatment. Similarly, cell migration (Figure 7c) and cell invasion (Figure 7d) of TS-treated NA were significantly repressed by emodin treatment, reinforcing the notion that emodin is capable of suppressing various malignant features of NA cells imposed by recurrent EBV reactivation.

### 2.7. Inhibition of EBV Reactivation by Emodin Decreases Tumor Growth In Vivo

**Figure 8:**
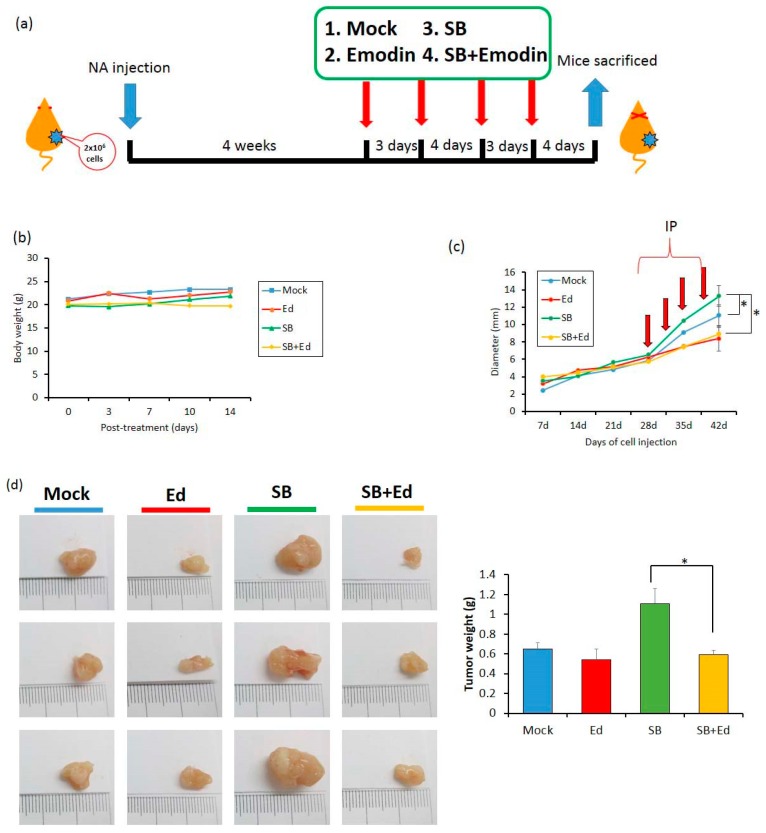
Emodin represses the tumor growth in a mouse model. NA cells were prepared for subcutaneous inoculation into severe combined immunodeficient (SCID) mice, which then received various treatments. (**a**) The diagram presents the schedule for in vivo assay of EBV reactivation inhibited by emodin. (**b**) The average animal body weights during the experiment were measured (*n* = 6 mice for each group). (**c**) The diameters of tumor nodules were monitored weekly using calipers throughout the experiment. (**d**) The tumor weights were measured after the mice were sacrificed. Tumor nodules were photographed as the listed pictures. Data are presented as mean ± SD. * *p* < 0.05.

To access the effect of emodin on EBV reactivation in vivo, two million NA cells were subcutaneously inoculated into the dorsal bodies of SCID mice. In our previous study, we found that it is too toxic if we administrated TPA plus SB to the mice and sole SB treatment is less toxic and is enough to reactivate the EBV harboring in NA cells in mice [26]. Approximately 4 weeks later, when the tumor size reached 0.5 cm, the mice were randomly assigned into four groups: mock, emodin, SB, and SB+emodin. Each compound was intraperitoneally administered into the mice every 3 or 4 days (Figure 8a). Of note, 0.6 mg/kg SB was sufficient to reactivate EBV lytic cycle and did not have a toxic effect in vivo (Figure 8b). After two weeks, the tumors were harvested for weighing and analysis. In the SB group, we found the average of tumor size increased after SB treatment, compared to the mock control (Figure 8c); however, this increase was repressed by emodin administration (Figure 8c). Meanwhile, the tumor size of the emodin group was similar to that of the mock control (Figure 8c). When we measured the tumor weights of the SB group, they were also heavier than those of the control group; however, the tumor weights were lower in the SB+emodin group (Figure 8d). Taken together, these results suggest that emodin has an inhibitory effect on tumor growth in the mouse model, via inhibition of EBV reactivation.

## 3. Discussion

NPC is a virus-associated cancer with a unique geographical and racial distribution. As one of the etiological agents of NPC, EBV has been marked as a therapeutic target for a long time. Large numbers of studies have focused on EBV latent proteins, including EBNA1, LMP1, and LMP2. At the present time, through the efforts of many scientists, the lytic cycle of EBV has been gradually unmasked to understand how it contributes to NPC tumorigenesis. We now know that the various EBV lytic proteins make different contributions to tumor progression, e.g., DNase and terminase cause genomic instability [20,22], Rta and BGLF4 induce chromosome aberration [19,37], BALF1 and BHRF1 exhibit an anti-apoptotic effect [16,21], and so on. Based on these findings, we can treat EBV reactivation as a target to develop the anti-cancer therapy and, moreover, several clinical trials to determine whether treatment with anti-EBV compounds could relieve the NPC tumorigenesis are in progress.

Recently, antimicrobial adjuvant therapies have become very popular for treating virus-related cancers and cancer-associated infections. EBV lytic replication-based therapies attract attention for cancer therapy and two treatment strategies are being developed vigorously. One is the inhibition of EBV reactivation to repress tumor growth and the other is the induction of EBV reactivation to enable cytolytic therapy [38]. Compared to the latter, the strategy of inhibiting the EBV lytic cycle has at least three benefits for anti-cancer therapy. First, blocking the EBV lytic cycle may stop the cell to cell transmission of EBV; second, inhibition of EBV reactivation could also reduce the expression of oncogenic lytic proteins and; third, suppressing EBV reactivation attenuates the oncogenic endocrine factors releasing after EBV enters the lytic cycle. These advantages have led to a massive search for new compounds, active against EBV lytic replication. Until now, many small molecules have been found to effectively suppress EBV lytic reactivation, especially natural compounds. Among the family of polyphenolics, EGCG attracts the most attention for anti-EBV activity and has been shown to inhibit EBV reactivation by repressing the MEK/ERK1/2 and PI3K/Akt pathways [33,39]. Curcumin has been found to inhibit EBV reactivation through interference with BZLF1 gene transcription [40]. Resveratrol has been found to have anti-EBV activity through suppression of the activation of the transcription factors NF-κB and AP-1 [41]. Sulforaphane is an anti-EBV agent that reduces the transactivation activity of Rta protein [42]. Among the family of flavonoids, luteolin has been shown to inhibit EBV reactivation by interfering with SP1 binding to Zp and Rp, and it represses tumor growth in a mouse model [25,26]. Genistein has anti-EBV activity through blocking the activation of IgG-mediated tyrosine kinase [43] and protoapigenone acts against EBV by reducing Zta transcriptional activity [44]. In addition to these two major families of natural compounds, andrographolide and moronic acid also have been reported to inhibit IE protein expression, with anti-EBV activity in the B-cell system [45,46]. Interestingly, most of these compounds are found in B-cell system in which the cellular environment may differ from the epithelial cells and may have less effect in an epithelial cell system. In fact, EBV prefers to remain latent in the B cells and progress to the lytic stage in epithelial cells. Thus, in our laboratory, we make an effort to search in the epithelial cell system for natural anti-EBV compounds for NPC therapy. Through preliminary screening, we found several compounds with anti-EBV activities and have reported some of them. In this study, we report another compound, emodin, which was found by our screening and has potential anti-EBV activity. We carried out immunoblotting (Figure 2), immunofluorescence assays (Figure 3a,b), and flow cytometric analysis (Figure 3c,d) to show that emodin treatment inhibits the expression of EBV lytic proteins. We also demonstrated that emodin decreases EB virion production in NPC cell lines. Further experiments indicate that emodin repressed the promoter activities of Zp and Rp (Figure 5). To dissect the underlying mechanism, the Z1D and ZII domains were shown to be involved in Zp inhibition by emodin, which may be through the downregulation of SP1 expression (Figure 6). Finally, we showed that the administration of emodin restrains the reactivation-induced tumorigenic properties and represses tumor growth in a mouse model. All of these results suggest that emodin has potential for EBV-based NPC therapy.

From another aspect, emodin also shows a good potential for anti-EBV therapy in epithelial (Figure 4) and B cell systems [36]. We also showed that emodin represses SP1 expression in epithelial cells, which is consistent with other reports [47,48]. It is known that binding of SP1 is crucial for the activation of several viruses; in fact, some of these viruses have been shown to be inactivated by emodin [33,49], implying a wide inhibitory spectrum.

Although we demonstrated that the emodin inhibits EBV reactivation by interfering with SP1 expression, this raises a further question: how does emodin affect SP1 expression? It is well-known that the expression of SP1 is highly dependent on the cell cycle. Regulation of SP1-dependent transcription is affected by several pathways, including the amount of SP1 protein, its DNA binding activity, and its transactivation activity. Through analyzing the composition of SP1 promoter, regulation of SP1 expression has been shown to depend on the relative amounts of SP1, SP3, E2F, and NF-Y proteins in the cell nucleus [50]. Because emodin is able to inhibit E2F expression [51], accordingly, one possible mechanism by which emodin inhibits SP1 expression is through inhibition of E2F expression. Moreover, overexpression of p53 also has been found to decrease sp1 mRNA [52] and emodin is able to upregulate the p53 protein level to arrest the cell cycle and induce apoptosis [53]. Hence, emodin treatment may inhibit SP1 expression by upregulation of p53. In addition, we might also reasonably postulate that emodin may interfere with SP1 expression via its anti-oxidative activity, because SP1 is upregulated in cells by oxidative stress [54].

In summary, for repression of reactivation-induced tumorigenesis, finding efficient compounds against EBV reactivation is an important issue. To this end, we screened and found that emodin possesses anti-EBV activity, which can be utilized in repressing tumor growth in vitro and in vivo. This study gives a new insight into the application of emodin and we hope it may provide an alternative choice for antiviral therapy and cancer prevention.

## 4. Materials and Methods

### 4.1. Reagents and Antibodies

The chemicals used in this study, including emodin, 12-*O*-tetradecanoyl-phorbol-1,3-acetate (TPA) and sodium butyrate (SB), were purchased from Sigma-Aldrich Co. (St. Louis, MO, USA). EBV antibodies used in this study are as follows: anti-EBV Zta 4F10 [55], anti-EBV Rta 467 (unpublished), anti-BMRF1 (EAD) 88A9 [56], anti-DNase 311H [55], and anti-GAPDH (Sigma-Aldrich Co.).

### 4.2. Cell Lines

TW01 and HONE-1 are NPC cell lines which were kind gifts from Dr. CT Lin [57] and Dr. R Glaser [58], respectively. NA and HA are EBV-infected NPC cell lines obtained from co-culture of rAkata cells and NPC cells, TW01 and HONE-1, respectively, and were selected by G418 treatment [59]. All NPC cell lines were maintained in DMEM (Dulbecco’s modified Eagle’s medium) with 10% fetal calf serum (FCS).

### 4.3. EBV Induction and Emodin Treatment

To determine whether emodin could induce EBV reactivation, the EBV-positive cell lines NA and HA were seeded for 24 h and then treated with various amounts of emodin for a further 24 h and harvested for analysis. To determine whether emodin could inhibit EBV reactivation, NA and HA cells were seeded for 24 h. Before induction, cells were pretreated with emodin for 1 h and then TPA (40 ng/mL) plus SB (3 mM) were added to induce EBV reactivation. After 24 or 48 h of incubation, cells were harvested for further analysis.

### 4.4. Evaluation of the Cytotoxicity of Emodin

The cytotoxicity of emodin to all cell line was determined by WST-1 assay (Invitrogen, Carlsbad, CA, USA), according to the manufacturer’s instructions. Briefly, two sets of cell lines, including NA and TW01 cells (5 × 10^3^ cells/well) and HA and HONE-1 cells (1 × 10^4^ cells/well), were seeded in 96 well plates. The cells were incubated with various concentrations of emodin (0, 1, 10, 20, 50, and 100 μM) for 48 h and the cytotoxicity was measured by WST-1 assay. The fluorescence was determined using a microplate reader (Infinite M200, Tecan, Männedorf, Switzerland). The CC_50_ value (half maximum of cytotoxicity concentration) was defined as the concentration of emodin which killed 50% of the cells. The mean and standard deviation were calculated from at least three independent experiments.

### 4.5. Western Blotting Analysis

The cell extracts were separated by 10% SDS-PAGE and transferred to Hybond-C super membrane (Amersham Biosciences Ltd., Little Chalfont, UK). The blots were first incubated with blocking buffer (10 mM Tris-HCl, pH 8.0, 0.9% NaCl, and 4% skim milk) for 1 h and then reacted with the antibodies for 1 h at room temperature. After washing three times with washing buffer (10 mM Tris-HCl, pH 8.0, 0.9% NaCl), the blots were incubated with horseradish peroxidase-labeled goat anti-mouse IgG (1:20,000; Amersham Biosciences Ltd.) with blocking buffer for 1 h at room temperature. After incubation overnight, the membranes were washed with washing buffer and then developed with a freshly prepared substrate (Amersham Biosciences Ltd.). The luminescence was detected using a short exposure to X-ray film. The original pictures of western blotting can be found in the Supplementary Materials (Figures S1–S3).

### 4.6. Immunofluorecence and Flow Cytometry Analysis

To determine the number of the cells which switched into the lytic cycle, immunofluorescence and flow cytometry analysis were carried out as in previous studies [20]. Briefly, for immunofluorescence staining, treated cells were fixed in 2% formaldehyde solution for 10 min. The fixed cells were permeabilized with 0.4% Triton X-100 in PBS for a further 5 min. After washing with 4% FCS in PBS, the cells were blocked in 4% FCS-PBS for 30 min and incubated with anti-EAD antibody (dilution 1:10) for 1 h at room temperature. The secondary antibody, rhodamine- conjugated goat antimouse IgG, which was diluted 1:100 in 4% FCS-PBS was added for 1 h. Finally, the cells were washed with 4% FCS-PBS and observed by fluorescence microscopy. The nuclei were stained with DAPI (1:10,000; Sigma-Aldrich Co.) staining. For flow cytometric analysis, treated cells were harvested and immediately fixed in 70% ethanol. The fixed cells were permeabilized with 1% Triton X-100 in 4% FBS and incubated with anti-EAD antibody (dilution 1:10) for a further 2 h. The cells were washed with PBS and incubated with a goat anti-mouse IgG rhodamine-conjugated antibody (1:1000) for 1 h. The cells were washed and were adjusted to a Becton Dickinson FACScan flow cytometer (BD Biosciences, San Jose, CA, USA). Each sample was tested with 10,000 cells.

### 4.7. Determination of the Copy Number of the EBV Genome

For detection of released EBV particles, NA cells (1 × 10^6^ cells/well) were incubated with TPA (40 ng/mL) and SB (3 mM) 48 h after pretreatment with emodin for 1 h. The supernatants were filtered through a 0.45 μM filter and then each

Supernatant (160 mL) was incubated with 2 mL DNase I and 10× DNase I buffer (10 mM Tris-HCl, 2.5 mM MgCl_2_, 0.5 mM CaCl_2_, pH 7.6) at 37 °C for 60 min, 20 mL of 2 mM EDTA (pH 8.0) was then added to terminate the DNase I activity. Each sample was then treated with 0.1 mg/mL proteinase K (sample: proteinase K = 1:1 [vol/vol]) at 50 °C for 60 min and the reactions were stopped by inactivating the proteinase K activity at 75 °C for 20 min. Subsequently, the samples were examined for the fragment of BALF5, the DNA polymerase of EBV, by real-time PCR analysis (Chen et al. 2011) (sense: 5′-CGGAGTTGTTATCAAAGAGGC-3′; antisense: 5′-CGAGAAAGACGGAGA TGGC-3′). Each reaction mixture contained 2 mL prepared viral DNA from each sample, 7.5 mL master mix (Kapa Biosystems, Fast qPCR kit), 0.3 mL specific primers (0.2 mM), and PCR-grade water to a final volume of 15 mL. The qPCR conditions were: 5 s denaturation at 95 °C, 20 s annealing at 60 °C, and 2 s extension of primers at 72 °C for 45 cycles. The specificity of the PCR reaction was controlled by melting curve analysis (65–95 °C, 0.1 °C/s) in the LightCycler 480 (Roche Applied Science, Indianapolis, IN, USA). The results from three independent experiments were used to calculate the mean and standard deviation.

### 4.8. Transfection and Analysis of Luciferase Reporter Activity

The construction of the Zp and Rp reporter plasmids have been described previously [25,27]. Zta and Rta plasmids and all reporters were transfected using Lipofectamine 2000 (Invitrogen, Carlsbad, CA, USA). Briefly, for chemical induction, NA or TW01 cells were seeded in the numbers of 2 × 10^5^ per well. The Zp or Rp plasmid, mixed with Lipofectamine 2000 (Invitrogen) in Opti-MEM medium (Invitrogen), was incubated for 20 min at room temperature, and then added to each well. After 3 h incubation, emodin was then added or not for pre-treatment in 1 h, and then TPA (40 ng/mL) plus SB (3 mM) were added to induce EBV into the lytic cycle. For Zta or Rta induction, Zp or Rp was co-transfected with the Zta-expressing or Rta-expressing plasmids into NA and TW01 cells, respectively. Emodin was then added after 3~4 h of transfection. After induction for 24 h, the cells were lysed with 50 μL HEPES buffer (0.1M HEPES, pH 7.8, 1% Triton X-100, 1 mM CaCl_2_ and 1 mM MgCl_2_) and 25 μL of the lysates were added to combine with 25 μL of Luciferase Assay Reagent II (Promega Corporation, Madison, WI, USA) for 10 min of incubation. Finally, the luciferase activity was detected using a luminescence counter (Packard Instrument Company, Meriden, CT, USA). Each lysate sample was standardized with the expression of β-actin to control the variation in different samples. The mean and standard deviation of each sample were calculated from three independent experiments in duplicate.

### 4.9. Determination of MN Formation

Detection of MN formation was performed as described previously [60]. Briefly, the cells under repeated treatment with inducers combined with or without emodin were seeded onto coverslips to adhere. After 24 h incubation, the culture medium was removed and the cells were washed twice with PBS (pH 7.4). The cells were fixed with ice-cold methanol immediately for 15 min. After washing with PBS, the cells were stained with DAPI (Sigma-Aldrich, St. Louis, MO, USA) for 15 min. Micronuclei were monitored using a fluorescence microscope (Olympus, Tokyo, Japan).

### 4.10. Cell Migration Assay

Cell migration assays were carried out using the Oris system (Platypus Technologies, Platypus Technologies, Madison, WI, USA) (Chiu et al. 2014). Briefly, the cells were seeded in 96-well plates with Oris stoppers for 24 h incubation. The stoppers were removed and the cells were incubated for a further 24 h to permit cell migration. The cells were then subjected to PI staining and photographed by fluorescence microscopy (Olympus, Tokyo, Japan). The closure of the cell-free zone was measured by Image J software (National Institute of Health, Bethesda, MD, USA). The cell migration is shown as the percentage of the closure, calculated using the following equation: [(pre-migration) area − (migration)area/(pre-migration)area] × 100. The mean and standard deviation of each sample were calculated from three independent experiments.

### 4.11. Cell Invasive Assay

Cell invasion assays were performed using HTS FluoroBlok inserts (Falcon, Cambridge, MA, USA), as described previously [20]. Briefly, the transwell membranes were coated with matrigel (Becton Dickinson, Franklin Lakes, NJ, USA). 1 × 10^5^ cells were seeded onto the matrigel-coated membranes in inserts with 2% FBS medium and the inserts were placed into 24-well plates with 10% FBS medium for 24 h incubation. After incubation, the membranes were fixed with methanol and stained with 50 μg/mL propidium iodide. The cells that invaded and transmigrated to the lower surface of the polycarbonate membrane were photographed using a fluorescence microscope and the cell numbers were counted and analyzed.

### 4.12. In Vivo Tumorigenesis Model

For in vivo tumorigenesis assays, SCID mice (six-week-old) were inoculated subcutaneously with NA cells (2 × 10^6^ cells) for 4 weeks. During this period, the tumor growth was observed. When growing to approximately 0.5 cm in diameter, the mice were separated into four groups, each containing six mice. The 1st group served as the mock control, the 2nd group received 40 mg/kg of emodin, the 3rd group received 0.6 mg/kg SB, and the 4th group received 40 mg/kg of emodin and 0.6 mg/kg SB. The compounds were delivered by IP injection every 3 or 4 days. The health of the mice and tumor sizes were monitored 3 or 4 days after treatment and the diameters of the tumors were measured by calipers. Two weeks later, the mice were sacrificed and the tumors were excised for weighing and analysis. This animal study was approved by the Institutional Animal Care and Use Committee (IACUC) of National Health Research Institutes, Taiwan. (Protocol No: NHRI-IACUC--104032-A).

## 5. Conclusions

For the purpose of screening efficient dietary compounds, we found that emodin is capable to inhibit EBV reactivation in epithelial cells. It not only represses the expression of EBV lytic proteins, but also blocks EBV viral particle production. The reporter assays revealed that it inhibits Zp and Rp activity through attenuating the protein expression of SP1. Based on its great anti-EBV activity, emodin treatment can inhibit the reactivation-elicited tumorigenic properties, including MN formation, cell proliferation, migration, matrigel invasiveness, even tumor growth in mice. We conclude that emodin is a promising compound for anti-viral and anti-cancer therapies, which provides a good alternative choice for therapy of EBV-related diseases and malignancies.

## Supplementary Materials

The following are available online at https://www.mdpi.com/2072-6694/11/11/1795/s1, Figure S1: The original western results and band densitometry by Image J of Figure 2a, Figure S2: The original western results and band densitometry by Image J of Figure 2b, Figure S3: The original western results and band densitometry by Image J of Figure 6c.
