# Supplementary material for: Emodin Inhibits EBV Reactivation and Represses NPC Tumorigenesis
Source: Cancers (Basel). 2019 Nov 15;11(11):1795. doi: 10.3390/cancers11111795 (PMC6896023; doi:10.3390/cancers11111795)
Supplement: Supplementary file 1 [file cancers-11-01795-s001.pdf]

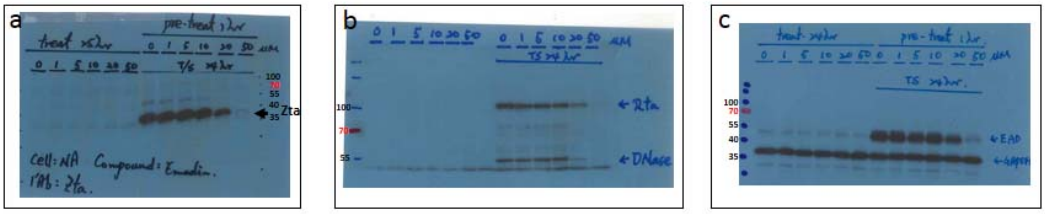

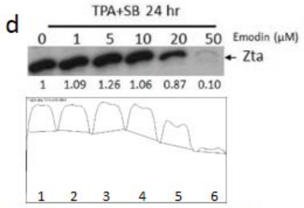

Band	Readings	Z/G	Ratio
1	6416.083	1.706344	1
2	6996.619	1.863566	1.092139
3	7642.104	2.151767	1.261039
4	7591.075	1.815247	1.063823
5	6044.125	1.489323	0.872815
6	933.941	0.178214	0.104442

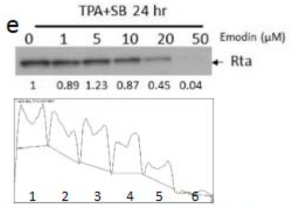

Band	Readings	R/G	Ratio
1	9644.832	2.565023	1
2	8577.066	2.284521	0.890644
3	11206.56	3.155402	1.230165
4	9382.69	2.243674	0.874719
5	4692.853	1.156358	0.450818
6	491.213	0.093733	0.036543

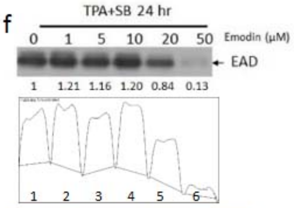

Band	Readings	EAD/G	Ratio
1	13313.125	3.540599	1
2	16097.56	4.287622	1.210988
3	14595.66	4.109661	1.160725
4	17739.296	4.241982	1.198097
5	12204.439	3.007275	0.849369
6	2438.74	0.465358	0.131435

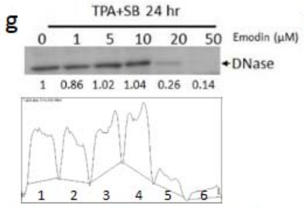

Band	Readings	DN/G	Ratio
1	12944.004	3.442432	1
2	11083.711	2.952172	0.857583
3	12451.116	3.505828	1.018416
4	14971.602	3.580146	1.040005
5	3605.095	0.888325	0.258052
6	2536.134	0.483942	0.140582

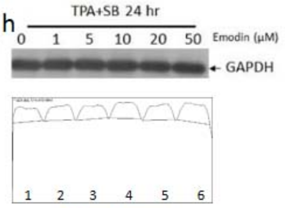

Band	Readings
1	3760.134
2	3754.426
3	3551.548
4	4181.841
5	4058.305
6	5240.569

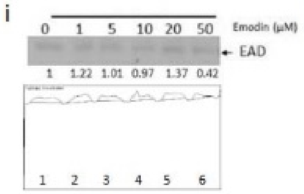

Band	Readings	EAD/G	Ratio
1	1397.577	0.107828	1
2	1332.648	0.131478	1.219327
3	982.627	0.109057	1.011397
4	1021.77	0.104098	0.96541
5	1262.355	0.147613	1.368963
6	499.335	0.045297	0.420088

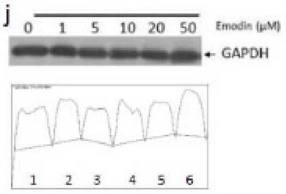

Band	Readings
1	12961.146
2	10135.933
3	9010.225
4	9815.439
5	8551.811
6	11023.518

Supplementary Figure 1. The original western results and band densitometry by Image J of Figure 2a.

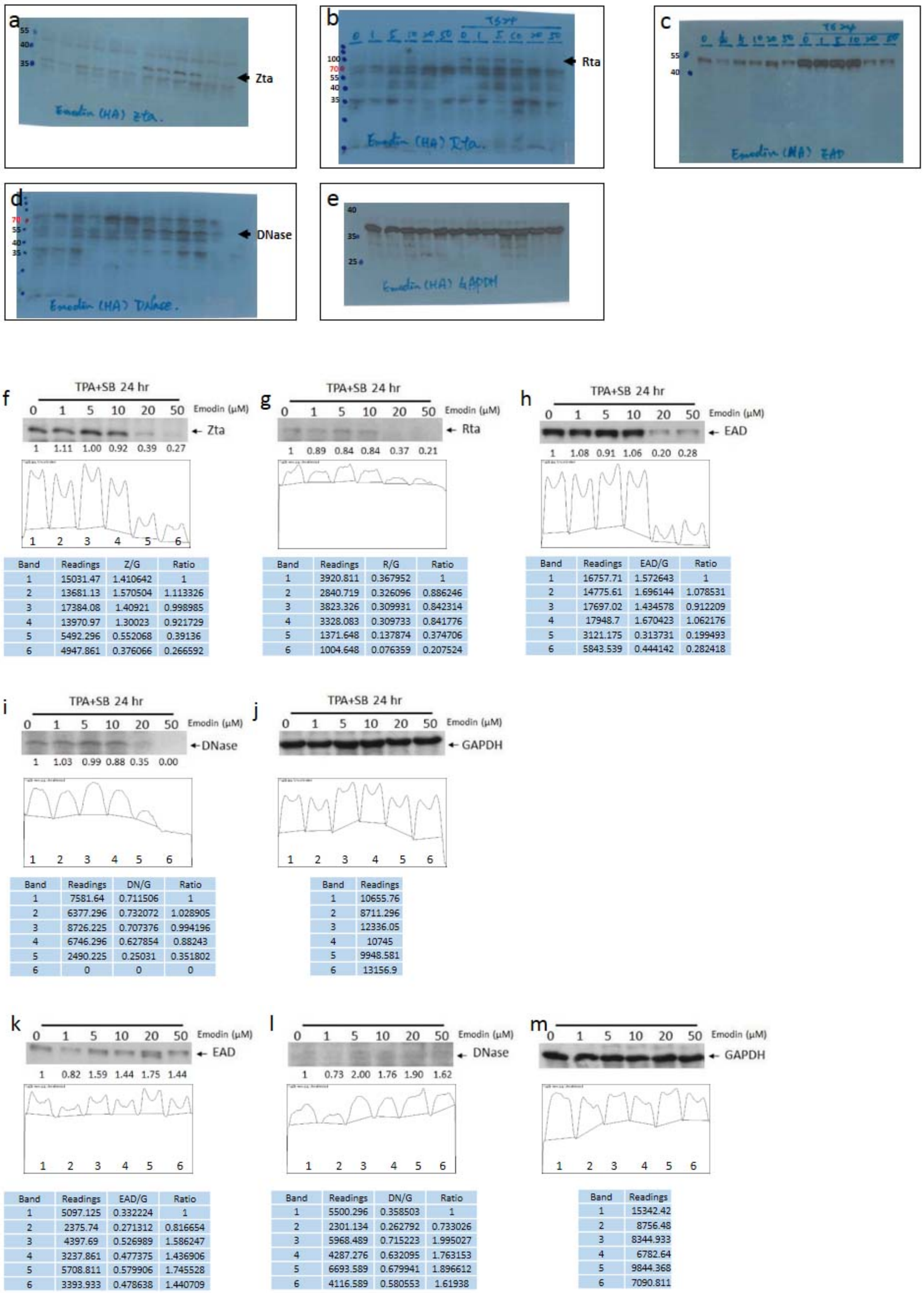

Band	Readings	Z/G	Ratio
1	15031.47	1.410642	1
2	13681.13	1.570504	1.113326
3	17384.08	1.40921	0.998985
4	13970.97	1.30023	0.921729
5	5492.296	0.552068	0.39136
6	4947.861	0.376066	0.266592

Band	Readings	R/G	Ratio
1	3920.811	0.367952	1
2	2840.719	0.326096	0.886246
3	3823.326	0.309931	0.842314
4	3328.083	0.309733	0.841776
5	1371.648	0.137874	0.374706
6	1004.648	0.076359	0.207524

Band	Readings	EAD/G	Ratio
1	16757.71	1.572643	1
2	14775.61	1.696144	1.078531
3	17697.02	1.434578	0.912209
4	17948.7	1.670423	1.062176
5	3121.175	0.313731	0.199493
6	5843.539	0.444142	0.282418

Band	Readings	DN/G	Ratio
1	7581.64	0.711506	1
2	6377.296	0.732072	1.028905
3	8726.225	0.707376	0.994196
4	6746.296	0.627854	0.88243
5	2490.225	0.25031	0.351802
6	0	0	0

Band	Readings
1	10655.76
2	8711.296
3	12336.05
4	10745
5	9948.581
6	13156.9

Band	Readings	EAD/G	Ratio
1	5097.125	0.332224	1
2	2375.74	0.271312	0.816654
3	4397.69	0.526989	1.586247
4	3237.861	0.477375	1.436906
5	5708.811	0.579906	1.745528
6	3393.933	0.478638	1.440709

Band	Readings	DN/G	Ratio
1	5500.296	0.358503	1
2	2301.134	0.262792	0.733026
3	5968.489	0.715223	1.995027
4	4287.276	0.632095	1.763153
5	6693.589	0.679941	1.896612
6	4116.589	0.580553	1.61938

Band	Readings
1	15342.42
2	8756.48
3	8344.933
4	6782.64
5	9844.368
6	7090.811

Supplementary Figure 2. The original western results and band densitometry by Image J of Figure 2b.

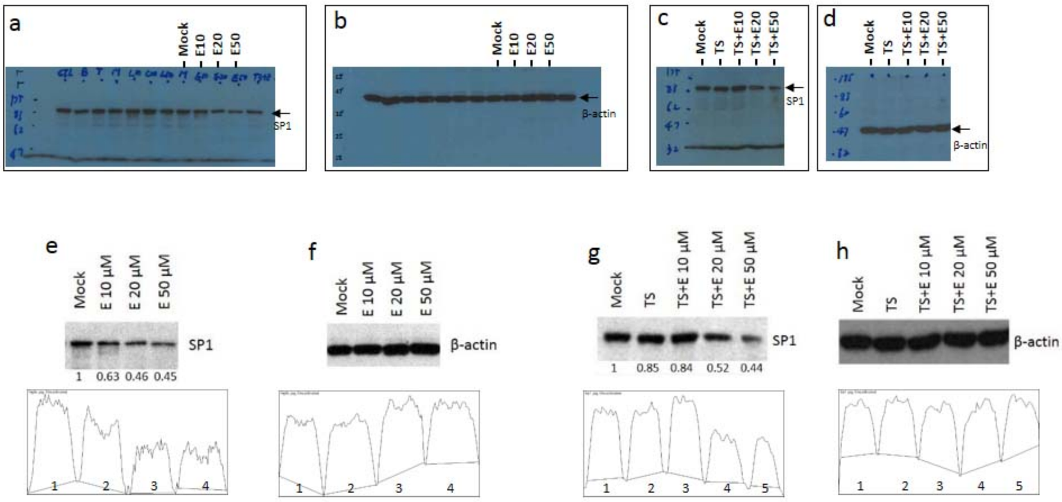

Band	Readings	SP1/act	Ratio
1	32399.89	1.318238	1
2	25469.64	0.831643	0.630875
3	17404.53	0.599934	0.455103
4	16550.43	0.596657	0.452617

Band	Readings
1	24578.18
2	30625.69
3	29010.74
4	27738.62

Band	Readings	SP1/act	Ratio
1	20795.8	1.284543	1
2	20166.34	1.093161	0.851012
3	22734.51	1.075787	0.837486
4	14265.15	0.672758	0.523734
5	9829.468	0.560223	0.436127

Band	Readings
1	16189.27
2	18447.73
3	21132.92
4	21203.97
5	17545.62

Supplementary Figure 3. The original western results and band densitometry by Image J of Figure 6c.
